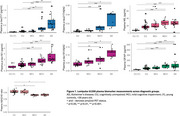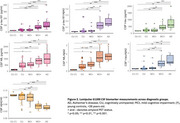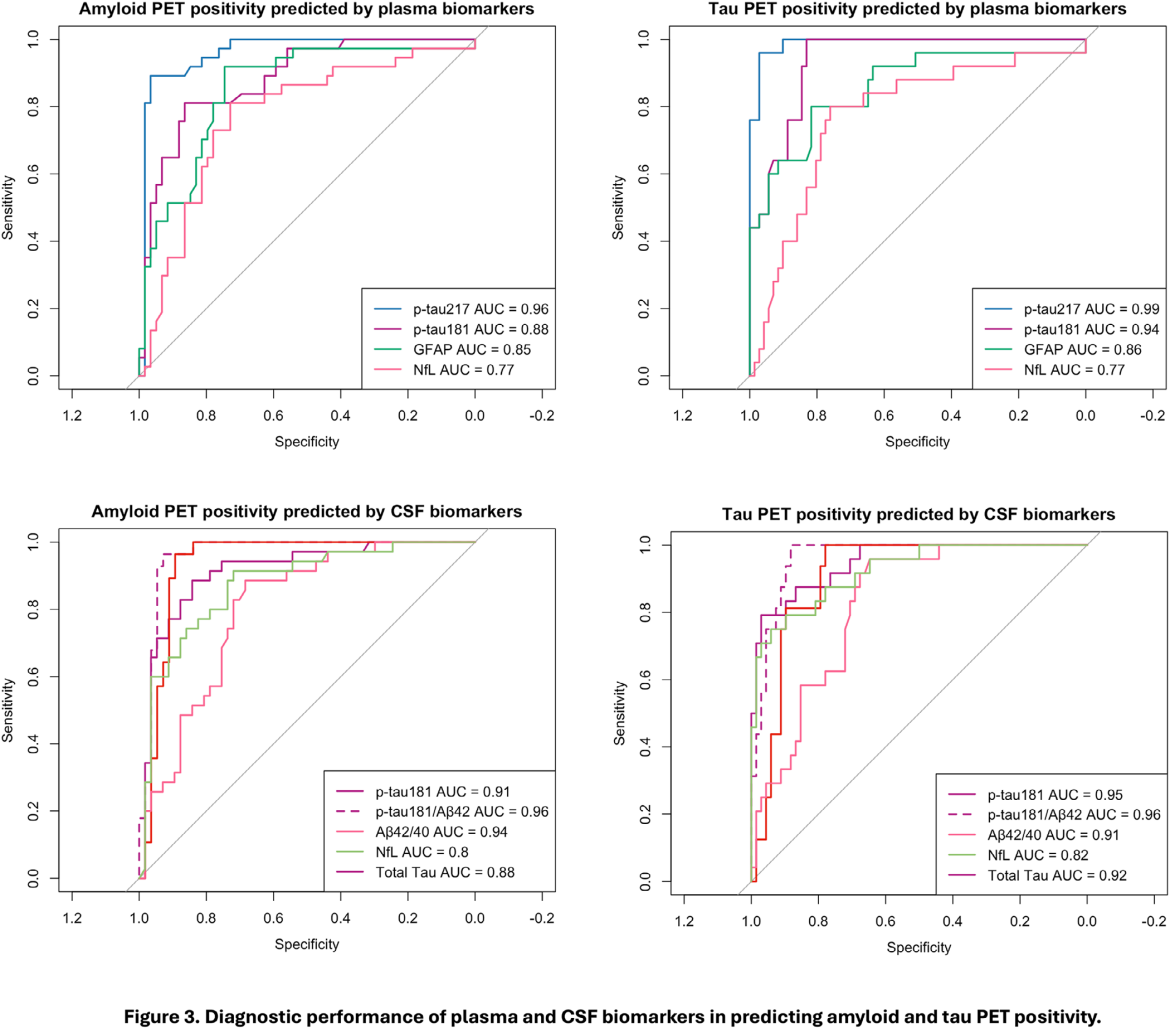# Clinical validation of Lumipulse G1200 automated immunoassays for Alzheimer's disease biomarkers in a Quebec cohort

**DOI:** 10.1002/alz70862_109923

**Published:** 2025-12-23

**Authors:** Tevy Chan, Nesrine Rahmouni, Yansheng Zheng, Marina P Gonçalves, Joseph Therriault, Arthur C. Macedo, Lydia Trudel, Kely Monica Quispialaya Socualaya, Seyyed Ali Hosseini, Brandon J Hall, Yi‐Ting Wang, Etienne Aumont, Jaime Fernandez Arias, Gleb Bezgin, Stijn Servaes, Robert Hopewell, Chris Hsiao, Paolo Vitali, Tharick A Pascoal, Henrik Zetterberg, Andrea Benedet, Pedro Rosa‐Neto

**Affiliations:** ^1^ Montreal Neurological Institute, Montreal, QC Canada; ^2^ McGill University, Montreal, QC Canada; ^3^ Translational Neuroimaging Laboratory, The McGill University Research Centre for Studies in Aging, Montréal, QC Canada; ^4^ Université du Québec à Montréal, Montréal, QC Canada; ^5^ The McGill University Research Centre for Studies in Aging, Montreal, QC Canada; ^6^ University of Pittsburgh, Pittsburgh, PA USA; ^7^ Departments of Psychiatry and Neurology, University of Pittsburgh School of Medicine, Pittsburgh, PA USA; ^8^ Institute of Neuroscienace and Physiology, University of Gothenburg, Mölndal, Västra Götaland Sweden; ^9^ University of Gothenburg, Gothenburg Sweden

## Abstract

**Background:**

With the anticipated arrival of disease‐modifying treatments for Alzheimer’s disease (AD) in Canada, integrating biomarkers into clinical practice is crucial to enhancing diagnostic accuracy and optimizing referrals for treatment. Lumipulse G1200 (Fujirebio) is a fully automated immunoassay instrument that streamlines the analysis of these biomarkers. In this study, we evaluated the diagnostic performance of Lumipulse G1200 plasma and CSF immunoassays in detecting AD pathology within a Quebec population cohort.

**Method:**

Plasma and CSF samples of 102 participants from the TRIAD cohort (median age 67 years, 54% female) were analysed. Kruskal‐Wallis with post hoc Benjamini‐Hochberg false discovery rate (BH) correction compared the levels of biomarkers among the diagnostic groups. Discriminative performance for Aβ (^18^F‐NAV4694) and tau (^18^F‐MK6240) PET status was assessed using the area under the curve (AUC) of receiver operating characteristic (ROC). Amyloid PET global SUVR > 1.55 and tau PET metaROI SUVR>2.5STD of the young controls determined Aβ and Tau PET positivity, respectively. Spearman’s correlation examined the association between plasma *p*‐tau217 and *p*‐tau181 with amyloid and tau PET SUVR.

**Result:**

Plasma *p*‐tau181 and *p*‐tau217 were higher in individuals with clinical diagnosis of AD compared to the cognitively unimpaired (CU) or MCI not due to AD (MCI‐). Plasma *p*‐tau217 very strongly correlated with both Aβ and tau PET SUVR (ρ=0.805 and 0.797 respectively, *p*‐value<2.2e‐16), as compared to plasma *p*‐tau181 (ρ=0.629 and 0.644 respectively, *p* < 4.097e‐10). Both assays identified with comparable high accuracy elevated Aβ pathology (plasma *p*‐tau217, AUC, 0.96, 95% CI: 0.92‐1.00; *p*‐tau181, AUC 0.88, CI 0.81‐ 0.96). However, plasma *p*‐tau217 had higher discriminative performance than *p*‐tau181 for tau PET (*p*‐tau217, AUC 0.99, CI: 0.98‐1.00; *p*‐tau181, AUC 0.94, CI 0.89‐0.98, DeLong’s test *p*‐value<0.01). CSF *p*‐tau181, *p*‐tau181/Aβ42 and Aβ42/40 had excellent discriminative performance for Aβ and tau PET positivity. Moreover, plasma *p*‐tau217 had similar performance as CSF *p*‐tau181 for predicting Aβ and tau PET positivity.

**Conclusion:**

Lumipulse G1200 immunoassays showed excellent agreement with amyloid and tau PET. Their ease of use and high diagnostic accuracy make them strong candidates for clinical implementation.